# The Diverse Distribution of Risk Factors between Breast Cancer Subtypes of ER, PR and HER2: A 10-Year Retrospective Multi-Center Study in China

**DOI:** 10.1371/journal.pone.0072175

**Published:** 2013-08-20

**Authors:** Qingkun Song, Rong Huang, Jing Li, Jinhu Fan, Shan Zheng, Bin Zhang, Hongjian Yang, Zhonghua Tang, Jianjun He, Xiaoming Xie, Hui Li, Jiayuan Li, Youlin Qiao

**Affiliations:** 1 Departments of Epidemiology, Pathology, Cancer Institute & Hospital, Chinese Academy of Medical Sciences & Peking Union Medical College, Beijing, China; 2 Department of Epidemiology, West China School of Public Health, Sichuan University, Chengdu, China; 3 Department of Breast Surgery, Liaoning Cancer Hospital, Shenyang, China; 4 Department of Breast Surgery, Zhejiang Cancer Hospital, Hangzhou, China; 5 Department of Breast-thyroid Surgery, Xiangya Second Hospital, Central South University, Changsha, China; 6 Department of Oncosurgery, The First Affiliated Hospital of Medical College, Xi’an JiaoTong University, Xi’an, China; 7 Department of Breast Oncology, Sun Yat-Sen University Cancer Center, Gungzhou, China; 8 Department of Breast Surgery, The Second People’s Hospital of Sichuan Province, Chengdu, China; The University of Texas MD Anderson Cancer Center, United States of America

## Abstract

**Introduction:**

Hormone receptors, human epidermal growth factor receptor 2 and some risk factors determine therapies and prognosis of breast cancer. The risk factors distributed differently between patients with receptors. This study aimed to investigate the distribution of risk factors between subtypes of breast cancer by the 3 receptors in Chinese native women with a large sample size.

**Methods:**

The multi-center study analyzed 4211 patient medical records from 1999 to 2008 in 7 regions of China. Data on patients’ demographic information, risk factors (menopausal status, parity, body mass index) and receptor statuses were extracted. Breast cancer subtypes included ER (+/−), PR (+/−), HER2 (+/−), 4 ER/PR and 4 molecular subtypes. *Wilcoxon* and *Chi-square* tests were used to estimate the difference. The unconditional logistic regression model was used for analysis, and presented *p-value* after *Bonferroni* correction in the results.

**Results:**

Compared to patients with negative progesterone receptor, the positive patients were younger at diagnosis, and reported less likely in postmenopausal status and lower parity (*p<0.05*). Comparing with the subtype of ER+/PR+, ER+/PR− subtype were 4-year older at diagnosis (OR = 1.02), more likely to be postmenopausal (OR = 1.91) and more likely to have >1 parity (OR = 1.36) (*p<0.05*); ER−/PR− subtype were more likely to be postmenopausal (OR = 1.33) and have >1 parity (OR = 1.19) (*p<0.05*). In contrast to the luminal A subtype, triple negative subtype had a lower BMI (OR = 0.96) and ORs of overweight and obesity reduced by >20% (*p<0.05*).

**Conclusion:**

In this study, it was found that Chinese female patients did have statistically significant differences of age, menopausal status, parity and body mass index between breast cancer subtypes. Studies are warranted to further investigate the risk factors between subtypes, which was meaningful for prevention and treatment among Chinese females.

## Introduction

Breast cancer (BC) is a significant threat to women’s health in China. In 2008, 170 000 new cases occurred, making it the first cause of cancer deaths in Chinese females [1]. The age-standardized rate of incidence was 21.6/10^5^ and had an increasing trend which suggested the serious challenge [1]. On the other hand, it is well established that BC can have a favorable survival rate: the overall 5-year relative survival rate is 89.0% [2]. Because of high incidence but relatively effective prognosis, BC becomes the most prevalent cancer worldwide [3].

Factors that have impact on treatment options and prognosis for BC include the immunohistochemical status of estrogen receptors (ER), progesterone receptors (PR) and the human epidermal growth factor receptor 2 (HER2) [4]. In combination with ER, PR and HER2, some risk factors also determine therapy selection and the prognosis [5]. Women from western countries had a diverse range of body mass index (BMI) [6], reproductive factors [7] and other factors [4], [8] between subtypes. However, variations of risk factors between subtypes by receptors, especially molecular subtypes, have not been explored for Chinese women. To better understand the particular features and improve the treatment strategies for BC in China, there is a need to investigate the variations of risk factors between subtypes among Chinese women from different clinical centers, at a national level.

## Materials and Methods

### Ethics Statement

This study was approved by the Cancer Foundation of China’s Institutional Review Board. The institutional review board obtained written consent from participating hospitals to access patient medical records. As it was a 10-year retrospective design, some data subjects were deceased, and it was impossible to contact with the patients or their relatives so the Cancer Foundation of China waived the need for written informed consent from the participants.

### Methods

This was a 10-year retrospective multi-center analysis of 4211 female BC patients in China. 7 tertiary hospitals were selected-one from each district in Northeast, Southwest, Northwest, North China, East China, Central China and South China. The information of hospitals was listed in Table S1 and the tertiary hospitals from the 7 capital cities had the standardized techniques in receptor detection, cancer diagnosis and therapies. In each hospital, medical records of female inpatients from 1999 through 2008 were accessed. To do this, trained staff reviewed all files and selected patients under the three inclusion criteria, that: (1) there was pathological evidence of primary BC, (2) the patient’s admission date was within the randomly selected time frame(s) for each hospital, and (3) the patient received or was currently receiving treatment for BC (i.e., surgery, medical oncology, radiotherapy). The detailed description of the methods was reported in previous studies [9], [10].

From 1999 to 2008, there were 45200 female cases in the 7 centers, but for sampling frame, each year we only chose the patients in one month into the study. In 1999, a month was chosen randomly. In 2000, the subsequent month was used, and so on. The selected month each year in the 7 centers was listed in Table S2. Inpatients from the months of January and February were excluded because this is China’s traditional spring festival and individuals seldom visit hospitals. For example, in 1999, all patients in March from one hospital were enrolled, in 2000, the patients in April were enrolled and so on. In each selected month, if inpatient admissions were less than 50, cases from neighboring months were added until the total number reached 50. If inpatients’ number in the selected month exceeded 50, all cases in that month were chosen. In the end, there were 4211 cases in the 7 centers during 10-year period selected into the study.

Statuses of ER, PR and HER2 were collected from medical records in the 7 hospitals. In the 7 hospitals, the receptor status was all determined by immunohistochemistry, which was standardized and mutual recognition. In this study, positive HER 2 indicated overexpression and was classified by a score of 3+; negative HER 2 indicated HER2 having scores of 0, 1+ and 2+ [11]. Different combinations of ER or PR and HER2 defined the four molecular subtypes: (1) luminal A (ER+ or PR+ and HER2−), (2) luminal B (ER+ or PR+ and HER2+), (3) triple negative (TNBC) (ER−/PR− and HER2−), (4) and HER2+ (ER−/PR− and HER2+) [12].

The data analyzed included demographic information and risk factors. The defined risk factors included: age, BMI, menopausal status, age at menopause, age of first live birth, parity, breastfeeding status, and family history of BC. The collected data were patient information at diagnosis. Age meant the diagnosis age. BMI was estimated by weight in kilograms divided by squared height in meter. The classification of BMI was categorized using scales adapted for Asian adults - underweight (BMI<18.5 kg/m^2^), normal (18.5 kg/m^2^≤BMI <23 kg/m^2^), overweight (23 kg/m^2^≤BMI<25 kg/m^2^) and obese (BMI≥25 kg/m^2^) [13]. Family history of BC was defined as BC occurrence among the first-degree genetic relatives (i.e., parents, siblings, children). Positive breastfeeding status indicated the patients had a more than 6-month breastfeeding experience. More than 95% cases had no smoking, drinking history and hormone use, so they were not included in the analysis.

### Statistical Analysis

Statistical data analysis was performed using SAS 9.0 software (SAS Institute Inc. Cary, North Carolina). In the 2-group and multi-group comparisons, a *Wilcoxon* test was used for analysis of continuous and ordered categorical variables; *Chi-square* test was used to analyze unordered categorical variables. In 2-group comparisons, the variables with significant difference between subtypes were further adjusted by *Bonferroni* method that *p_bonferroni_* = *p* value×3 (times of comparisons for particular variable); in the multi-group comparisons, the significant variables were further analyzed in pairwise comparisons with *p-value* adjusted by *Bonferroni* method. In the pairwise comparisons, ER+/PR+ [14] and luminal A [15], [16] were set as the referral group for the better prognosis, all other ER/PR and molecular subtypes compared with the two subtypes. The variables in the pairwise comparisons with *Bonferroni* adjusting *p-value* <0.05 were estimated crude odds ratios (OR) and 95% confidence intervals (95%CI) in unconditional logistic regression model. All the analyses were two-tailed tests with the significant level of 0.05.

## Results

### Subtype of Individual ER, PR and HER2

Among the study subjects, ER was positive in 2028 (57.4%) patients, PR was positive in 2058 patients (58.2%) and HER2 was positive in 736 cases (22.8%) (Table 1). Between ER+ and ER− cases, there was no significant difference of risk factors; between positive and negative HER2 cases, there was no significantly different distribution either (Table 1). Compared to PR− cases, PR+ cases were 2-year younger at diagnosis (*p*<0.05), less likely to be post menopausal by 9.1% (*p*<0.05), and less likely to have >1 parity by 6.6% (*p*<0.05) (Table 1).

**Table 1:** The characteristics of risk factors in subtypes of breast cancer.

	ER		*p*	PR		*p*	HER2		*p*
	Positive (n = 2028)	Negative (n = 1506)		Positive (n = 2058)	Negative (n = 1476)		Positive (n = 736)	Negative (n = 2495)	
Median age*	48.0	48.0	0.48	47.0	49.0	**0.002** #	48.0	48.0	0.81
Median BMI (kg/m^2^)*	23.1	22.8	0.10	23.1	22.8	0.10	23.3	22.92	0.12
Classification of BMI (kg/m2)*			0.06			0.06			0.21
<18.5	56 (3.3)	55 (4.5)		62 (3.6)	49 (4.1)		20 (3.0)	83 (3.9)	
<23.0	779 (45.6)	576 (47.3)		781 (44.9)	574 (48.3)		300 (44.8)	1001 (46.4)	
<25.0	376 (22.0)	257 (21.1)		391 (22.5)	242 (20.4)		148 (22.1)	463 (21.5)	
≥25.0	499 (29.2)	329 (27.0)		505 (29.0)	323 (27.2)		201 (30.0)	609 (28.3)	
Menopausal status&			0.27			**<0.001** #			0.78
Pre-menopause	1281 (63.2)	924 (61.4)		1362 (66.2)	843 (57.1)		460 (62.5)	1545 (61.9)	
Post-menopause	747 (36.8)	582 (38.7)		696 (33.8)	633 (42.9)		276 (37.5)	950 (38.1)	
Median age at menopause*	50.0	50.0	0.81	50.0	50.0	0.50	50.0	50.0	0.67
Median age of first living birth*	25.0	25.0	0.12	25.0	25.0	0.15	25.0	25.0	0.25
Parity*			0.17			**0.002** #			0.62
0	51 (2.7)	27 (1.9)		55 (2.8)	23 (1.7)		9 (1.3)	62 (2.6)	
1	961 (49.9)	690 (48.3)		1000 (51.0)	651 (46.6)		343 (49.4)	1168 (49.2)	
>1	915 (47.5)	713 (49.9)		905 (46.2)	723 (51.8)		342 (49.3)	1145 (48.2)	
Breastfeeding&			0.30			0.22			0.58
Yes	1250 (90.2)	912 (91.4)		1266 (90.1)	912 (91.6)		465 (91.4)	1533 (90.6)	
No	136 (9.8)	87 (8.6)		139 (9.9)	84 (8.4)		44 (8.6)	160 (9.5)	
Family BC history&			0.35			0.12#			>0.05#
No	1924 (96.5)	1414 (95.9)		1957 (96.8)	1381 (95.5)		690 (97.6)	2353 (95.6)	
Yes	69 (3.5)	60 (4.1)		64 (3.2)	65 (4.5)		17 (2.4)	108 (4.4)	

* Wilcoxon test;

& Chi-square test;

# p-value was Bonferroni adjusted.

### ER/PR Subtypes

BC cases had different distribution of age at diagnosis, menopausal status and parity between ER/PR subtypes (Table 2). With *Bonferroni* adjustment in the pairwise comparisons with ER+/PR+ subtype, ER+/PR− subtype was 3-year older at diagnosis (*p*<0.001), and had a 15.7% higher proportion in postmenopausal status (*p*<0.001) and a 8.1% higher proportion of >1 parity (*P = 0.0*24). ER−/PR− subtype had the The proportion of postmenopausal status and >1 parity in ER−/PR− subtype was 6.6% and 4.8% higher than ER+/PR+ subtype (*p*<0.05) (Table 2). Compared with ER+/PR+, ORs of 1-year increase of age at diagnosis, postmenopausal status and >1 parity were 1.02 (95%CI 1.01, 1.03), 1.91 (95%CI 1.51, 2.42) and 1.36 (95%CI 1.07, 1.74) in ER+/PR− subtype respectively; ORs of postmenopausal status and >1 parity were 1.33 (95%CI 1.14, 1.55) and 1.19 (95%CI 1.02, 1.39) in ER−/PR− subtype (Table 3).

**Table 2:** The characteristics of combination of ER and PR in breast cancer*.

	ER+/PR+ (n = 1691)	*P*	ER+/PR− (n = 337)		ER−/PR+ (n = 367)		ER−/PR− (n = 1139)	
			N (%)	*p_bonferroni_*	N (%)	*p_bonferroni_*	N (%)	*p_bonferroni_*
Median age&	47.0	**<0.001**	51.0	**<0.001**	46.0	0.16	48.0	0.39
Median BMI (kg/m^2^)&	23.1	0.12	23.0		23.2		22.7	
Classification of BMI (kg/m^2^)&		0.14						
<18.5	48 (3.3)		8 (2.9)		14 (4.6)		41 (4.5)	
<23.0	651 (45.3)		128 (46.9)		130 (43.1)		446 (48.7)	
<25.0	321 (22.3)		55 (20.2)		70 (23.2)		187 (20.4)	
≥25.0	417 (29.0)		82 (30.0)		88 (29.1)		241 (26.3)	
Menopausal status#		**<0.01**		**<0.001**		0.99		**0.001**
Pre-menopause	1112 (65.8)		169 (50.2)		150 (68.1)		674 (59.2)	
Post-menopause	579 (34.2)		168 (49.9))		117 (31.9)		465 (40.8)	
Median age at menopause&	50.0	0.77	50.0		50.0		50.0	
Median age of first living birth&	25.0	0.29	25.0		25.0		24.0	
Parity&		**0.01**		**0.024**		0.99		**0.04**
0	46 (2.9)		5 (1.6)		9 (2.6)		18 (1.7)	
1	823 (51.0)		138 (44.1)		177 (51.2)		513 (47.3)	
>1	745 (46.2)		170 (54.3)		160 (46.2)		553 (51.0)	
Breastfeeding#		0.54						
Yes	1032 (89.8)		218 (92.0)		234 (91.4)		694 (91.4)	
No	117 (10.2)		19 (8.0)		22 (8.6)		65 (8.6)	
Family BC history#		0.15						
No	1609 (96.9)		315 (94.6)		348 (96.4)		1066 (95.8)	
Yes	51 (3.1)		18 (5.4)		13 (3.6)		47 (4.2)	

* *p_bonferroni_* = 3×*p-value* in comparisons with subtype of ER+/PR+;

& *Wilcoxon* test;

# *Chi-square* test.

**Table 3:** Odds ratios (95%CI) between subgroups of combined ER and PR for breast cancer*.

	ER+/PR−	ER−/PR+	ER−/PR−
Median age	**1.02 (1.01, 1.03)**	0.99 (0.98, 1.00)	1.00 (1.00, 1.01)
Menopausal status			
Premenopausal	1.00	1.00	1.00
Postmenopausal	**1.91 (1.51, 2.42)**	0.90 (0.71, 1.14)	**1.33 (1.14, 1.55)**
Parity			
0	0.65 (0.25, 1.66)	0.91 (0.44, 1.89)	0.63 (0.36, 1.10)
1	1.00	1.00	1.00
>1	**1.36 (1.07, 1.74)**	1.00 (0.79, 1.26)	**1.19 (1.02, 1.39)**

* subtype of ER+/PR+ as the reference group.

### Molecular Subtypes

The percentages of luminal A, luminal B, HER2+ and TNBC subtypes were 54.5%, 14.0%, 8.8% and 22.7%, respectively. BMI, menopausal status and family history of BC were found to be different between the molecular subtypes (*p*<0.05) (Table 4). In the pairwise comparison to luminal A, the subtype of TNBC was more likely to have lower BMI (*p*<0.05) (Table 4), and OR of overweight and obesity decreased by more than 20% (*p*<0.05) (Table 5).

**Table 4:** Characteristics between subtypes of breast cancer combining ER, PR and HER2*.

	Luminal A (n = 1761)	*P*	Luminal B (n = 451)		HER2+ (n = 285)		TNBC (n = 734)	
			N (%)	*p_bonferroni_*	N (%)	*p_bonferroni_*	N (%)	*p_bonferroni_*
Median age&	48.0	0.06	47.0		50.0		48.0	
Median BMI& (kg/m^2^)	23.1	**0.01**	23.1	0.99	23.4	0.99	22.6	**0.01**
Classification of BMI& (kg/m2)		**0.01**		0.99		0.99		**0.006**
<18.5	53 (3.5)		12 (2.9)		8 (3.1)		30 (4.9)	
<23.0	686 (44.6)		189 (46.1)		111 (42.9)		315 (50.9)	
<25.0	346 (22.5)		88 (21.5)		60 (23.2)		117 (18.9)	
≥25.0	452 (29.4)		121 (29.5)		80 (30.9)		157 (25.4)	
Menopausal status#		**<0.05**		0.30		0.12		0.99
Pre-menopause	1101 (62.5)		301 (66.7)		159 (55.8)		444 (60.5)	
Post-menopause	660 (37.5)		150 (33.3)		126 (44.2)		290 (39.5)	
Median age at menopause&	50.0	0.38	50.0		50.0		50.0	
Median age of first living birth&	25.0	0.25	25.0		24.0		25.0	
Parity&		0.08						
0	48 (2.9)		7 (1.7)		2 (0.7)		14 (2.0)	
1	832 (49.7)		222 (52.2)		121 (45.0)		336 (48.0)	
>1	795 (47.5)		196 (46.1)		146 (54.3)		350 (50.0)	
Breastfeeding#		0.95						
Yes	1093 (90.5)		2901 (91.2)		175 (91.6)		440 (90.7)	
No	115 (9.5)		28 (8.8)		16 (8.4)		45 (9.3)	
Family BC history#		**<0.05**		0.18		0.99		0.52
No	1669 (96.0)		426 (97.9)		264 (97.1)		684 (94.7)	
Yes	70 (4.0)		9 (2.1)		8 (2.9)		38 (5.3)	

* *p_bonferroni_* = 3×*p-value* in comparisons with subtype of ER+/PR+;

& *Wilcoxon* test;

# *Chi-square* test.

**Table 5:** Odds ratios (95%CI) of various factors in subtypes of combined status of ER, PR and HER2*.

	TNBC	HER2+	Luminal B
Median BMI (kg/m^2^)	**0.96 (0.93, 0.99)**	1.02 (0.97, 1.06)	1.00 (0.97, 1.04)
Classification ofBMI (kg/m2)			
<18.5	1.22 (0.77, 1.95)	0.93 (0.43, 2.00)	0.83 (0.43, 1.58)
<23.0	1.00	1.00	1.00
<25.0	**0.73 (0.57, 0.94)**	1.07 (0.76, 1.50)	0.93 (0.70, 1.24)
≥25.0	**0.76 (0.60, 0.95)**	1.09 (0.80, 1.49)	0.98 (0.75, 1.27)

* subtype of luminal A as the reference group.

## Discussion

This study analyzed female BC patients from 1999 to 2008 at 7 geographic regions in China. In total, medical records of 4211 cases were used, with more than 75% having pathological testing of receptor status. Many factors distributed differently between BC subtypes by receptors. The diverse distribution of risk factors between BC subtypes has been reported in many studies among Western women. For Chinese native women, this study was the first to present such findings, providing a valuable reference for forward investigation.

### Menopausal Status

Shanghai Breast Cancer study reported similar results that more female patients at postmenopausal status were in subtypes of PR−, ER+/PR− and ER−/PR− [17]. In pre-menopausal status, the circulating steroid level was much higher, promoting the development of hormone-receptor positive BC and more positive PR cases occurred during this period. The cases with negative PR were more likely to happen in postmenopausal period. As molecular subtypes, Carolina Breast Cancer Study found similar results that no difference of postmenopausal status between luminal A and other molecular subtypes [4], but Devi et al. has previously reported a higher proportion of postmenopausal status in TNBC subtype than luminal A in Asian BC patients [18]. Race variations might explain this controversy. In this study, less than 50% of the cases were in postmenopausal status, as the Shanghai study [17], but in United States, the figure was much higher, around 80% [19]. BC incidence also had racial variation by molecular subtypes [20]. In China, the expression of ER, PR and HER2 in women were found to be different from women in the United States and Europe [10]. Chinese BC patients were found to have earlier ages of diagnosis and more proportion of positive HER2 [9], [10]. Even compared to other Asian races, Chinese female patients also had particular features in menopausal status and pathological features [4], [18]. These differences between races provided the possibilities of inconsistent results in studies. Further studies are required to explore the detailed mechanisms and racial differences in subtypes, as these differences may have effect on BC diagnosis.

### Age of First Live Birth

First live birth initiated the cellular differentiation for mammary gland [21] and the earlier differentiation induced lower susceptibility to carcinogenesis [22]. Therefore younger age of first live birth was a protector for BC. However, between BC subtypes no significant difference of age of first live birth was observed, which was also reported from a systematic review [23]. Oppositely, the Shanghai Breast Cancer Study [17] and another case-case analysis [7] presented younger age of first live birth in PR+ cancers than PR− ones. The inconsistent results needed further investigation in future studies.

### Parity

Full-term pregnancy started the differentiation of mammary gland cells and every new pregnancy might differentiate the undifferentiated cells, which reduced the susceptibility to carcinogenesis [22], [24]. Parous women had lower risk for BC than nulliparous women. The beneficial effect of parity was significant for women with positive PR, but not negative PR [23]. PR positive cases had less parity than PR negative cases, which was also reported from a pooled analysis [7]. The patients of ER−/PR− subtype having higher parity than ER+/PR+ subtype was reported from the pooled analysis [7] and other studies [19], [23], [25] too. However, in the Shanghai Breast Cancer Study, no difference of parity existed between ER/PR subtypes [17]. The inconsistency could possibly be the results of missing receptor status of some subjects in this study, the association needing further investigation.

### Family BC History

This study found no difference in family history of BC between subtypes, similar results also observed in other studies [7], [8]. Family BC history in the first-degree relatives increased the risk of all subtypes of BC, because of genetic susceptibility [17], [19]. But between the 4 ER/PR subtypes, the frequency of family BC history did not distributed differently [17].

### BMI

Among postmenopausal women, higher BMI increases the level of circulating steroids [26], [27] while reducing level of sex hormone-binding globulin [28], which increase the level of bioavailable estrogen and promote the development of hormone receptor-positive BC [17], [23], [29]. Patients of TNBC having lower BMI in our study was discrepant from the pooled analysis that patients of TNBC had a higher BMI (OR = 1.80, 95%CI 1.42, 2.29) than luminal A [7]. Among other Asian population, BMI was similar between molecular subtypes (*p*>0.05) [18]. The racial differences in BMI and BC subtypes [7], [18], s might explain the inconsistent results between Chinese people and others. The 4 molecular subtypes had different BMI value might indicate the particular etiology of BC for Chinese women, suggesting the need of further investigation.

Since BC subtypes had differences in median age, menopausal status, parity and BMI, further studies were possible to obtain a deceived result if they analyzed the cases without stratification by subtypes. The diverse frequency of risk factors between subtypes might indicate the independent etiology and therapeutic features. In addition, National Cancer Institute proposed that prognosis and selection of therapy may be influenced by the clinical and pathology features, such as the age, menopausal status, ER/PR status, and HER2 overexpression [30]. Therefore, receptors and some risk factors were the critical elements in preventive strategies, as well as treatment options for Chinese females.

The potential limitations of this study could have effect on results, although they are minimal. One tertiary hospital selected from one geographic region maybe the deficiency for good representative. But the tertiary hospitals had the standardized procedure and quality control for BC diagnoses, especially for pathological detection and laboratory tests. Sampling method was another suspected drawback of undermining data representativeness, which excluded 80% of prevalent patients, though it was the multi-center clinical study with largest sample size in China. Another limitation was a fraction of cases missing data in the study, possibly reducing the representativeness too. In these cases, case-case analysis was impossible to explore the risk factors for cancer, but was still useful in describing the difference of risk factors between subtypes.

## Conclusions

Age, menopausal status, parity and BMI were found to have statistically different distribution between BC subtypes by hormone receptors. Between molecular subtypes, TNBC had lower BMI than luminal A. BC subtypes did have diverse distribution in risk factors. The differences indicate that further prevention research should focus on subtypes individually and suggest the need for evidence supporting individual-based management for clinical treatment of BC.

## Supporting Information

Table S1 **Hospital information in the 7 regions.**
(DOCX)

Table S2 **The selected month in hospitals from 1999 to 2008.**
(DOCX)
