# Supplementary material for: The Diverse Distribution of Risk Factors between Breast Cancer Subtypes of ER, PR and HER2: A 10-Year Retrospective Multi-Center Study in China
Source: PLoS One. 2013 Aug 20;8(8):e72175. doi: 10.1371/journal.pone.0072175 (PMC3748061; doi:10.1371/journal.pone.0072175)
Supplement: Table S1 — Hospital information in the 7 regions. (DOCX) [file pone.0072175.s001.docx]

| Table S1. Hospital information in the 7 regions | | | |
| --- | --- | --- | --- |
| Hospital name | Region | City | Hospital level |
| Cancer Institute & Hospital, Chinese Academy of Medical Sciences | North China | Beijing | Tertiary |
| Liaoning Cancer Hospital | Northeast | Shenyang | Tertiary |
| Zhejiang Cancer Hospital | East China | Hangzhou | Tertiary |
| Xiangya Second Hospital | Central China | Changsha | Tertiary |
| the First Affiliated Hospital of Medical College, Xi’an JiaoTong University | Northwest | Xi'an | Tertiary |
| Sun Yat-Sen University Cancer Center | South China | Guangzhou | Tertiary |
| the Second People’s Hospital of Sichuan Province | Southwest | Chengdu | Tertiary |
