# Supplementary material for: The Diverse Distribution of Risk Factors between Breast Cancer Subtypes of ER, PR and HER2: A 10-Year Retrospective Multi-Center Study in China
Source: PLoS One. 2013 Aug 20;8(8):e72175. doi: 10.1371/journal.pone.0072175 (PMC3748061; doi:10.1371/journal.pone.0072175)
Supplement: Table S2 — The selected month in hospitals from 1999 to 2008. (DOCX) [file pone.0072175.s002.docx]

| Table S2. The selected month in hospitals from 1999 to 2008 | | | | | | | | | | |
| --- | --- | --- | --- | --- | --- | --- | --- | --- | --- | --- |
| City | Selected month in each year | | | | | | | | | |
|  | 1999 | 2000 | 2001 | 2002 | 2003 | 2004 | 2005 | 2006 | 2007 | 2008 |
| Beijing | 9 | 10 | 11 | 12 | 3 | 4 | 5 | 6 | 7 | 8 |
| Shenyang | 3 | 4 | 5 | 6 | 7 | 8 | 9 | 10 | 11 | 12 |
| Changsha | 6 | 7 | 8 | 9 | 10 | 11 | 12 | 3 | 4 | 5 |
| Guangzhou | 5 | 6 | 7 | 8 | 9 | 10 | 11 | 12 | 3 | 4 |
| Hangzhou | 7 | 8 | 9 | 10 | 11 | 12 | 3 | 4 | 5 | 6 |
| Xi'an | 4 | 5 | 6 | 7 | 8 | 9 | 10 | 11 | 12 | 3 |
| Chengdu | 8 | 9 | 10 | 11 | 12 | 3 | 4 | 5 | 6 | 7 |
